# Obituary Prof. Dr. med. Karl Köhle *1938 – †2022

**DOI:** 10.3205/zma001558

**Published:** 2022-07-15

**Authors:** Wolf Langewitz, Claudia Kiessling, Christoph Stosch

**Affiliations:** 1 Universitätsspital Basel, Switzerland; 2 Witten/Herdecke University, Faculty of Health, Education of Personal and Interpersonal Competencies in Health Care, Witten, Germany; 3 University of Cologne, Medical Faculty, Cologne, Germany

## Obituary

On 23 April 2022, Prof. Dr. med. Karl Köhle, one of the important protagonists of the last decades for a “speaking medicine” and pioneer of modern teaching and training in the field of clinical communication, passed away. 

Born in Munich in 1938, he spent a large part of his life in the Rhineland, but his love for Upper Bavaria and the region around Lake Starnberg remained throughout his life. After studying medicine and psychology in Hamburg, Munich and Vienna, he began his residency in 1967 in the Department of Internal Medicine and Psychosomatics under the direction of one of the pioneers of psychosomatic medicine in Germany, Prof. Thure von Uexküll, at the Centre for Internal Medicine and Paediatrics at Ulm University. In 1984 he was appointed Medical Director of the Clinic for Psychosomatics and Psychotherapy, University Hospital of Cologne, where he worked until his retirement in 2005. Karl Köhle has had a lasting influence on psychosomatic medicine in Germany through his person and his activities over many years.

**Figure 1:**
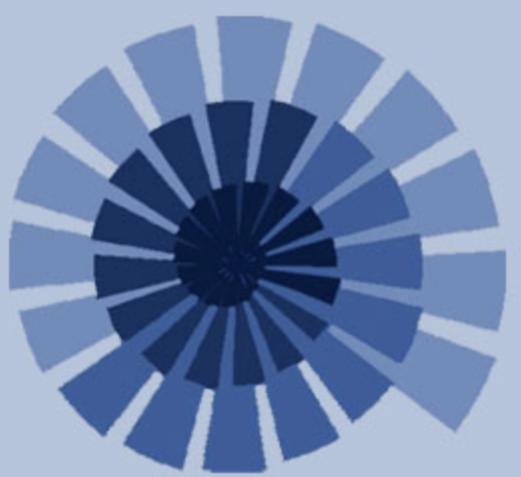
The teaching-learning spiral by Karl Köhle

Numerous innovations in the design of teaching can be traced back to Karl Köhle and his team, which still shape the discussion of communication skills training in Cologne today and at other faculties: as early as the end of the 1980s, he introduced problem-based learning into his courses as a means of dealing with the biopsychosocial model at the very beginning of the course of studies; at that time he already had in mind a longitudinal curriculum with a teaching-learning spiral (see figure 1 (Fig. 1)). In 1994, together with students on strike who had criticised the lack of orientation of medical studies towards the medical profession, he developed a history taking course as part of compulsory teaching. The use of simulated patients and work with videotaped patient consultations were established in the nineties. Karl Köhle thus made a significant contribution to the implementation of modern learning and assessment formats in Cologne and beyond, as well as to the promotion of communicative competence and psychosomatic concepts among medical students.

His research and teaching focused on clinical communication. He succeeded in getting to the heart of the essence of a conversation that gives a voice to those affected, defining concrete learning objectives and underpinning them with examples. In the technical implementation of this task, Karl Köhle was far ahead of his time. He was already using video-supported teaching when today’s common media were still the stuff of nerds’ dreams. For him, it was obvious that competences that show themselves in action would also have to be illustrated in action. A great team supported him in the development of NetmediaViewer. There were illustrative examples of real consultations, which were assigned to certain aspects of conversation and had their counterparts in basic chapters of “Der Uexküll” (textbook of psychosomatic medicine). Even in the early 2000s, the idea of using the NetMediaViewer in lectures and wanting to go on the internet during the lecture was so unusual, Wolf Langewitz remembers, that most of the people in charge of the lecture halls in Basel wanted to explicitly prevent this possibility so as not to distract students during the teaching. 

The long-standing cooperation with the family doctor Thomas Reimer was also trend-setting: the family doctor’s practice in the Eifel was connected to a group room in the Cologne University Hospital via a dedicated line. Students could follow the consultation when Dr Reimer spoke to patients in his practice. Afterwards, they could discuss their impressions with the family doctor and with an expert. There were video recordings of particularly impressive consultations, which documented the course of a medical history (and a history of illness) over a longer observation period. 

In the editorial team of “Der Uexküll”, textbook of Psychosomatic Medicine, Karl Köhle was the one who, as early as the 6^th^ edition in 2003, suggested to the publisher that a library of accompanying material accessible via the internet be created in the background (e.g. illustrative videos, lectures by Thure von Uexküll, etc.). His innovative drive was tireless and he was always able to inspire staff with his ideas, for example, an interactive presentation in which students could choose between different continuations at significant points in a conversation and then, controlled by an algorithm, ended up at completely different end points: E-learning avant la lettre!

The amazing thing about all this was the pragmatic approach to the topic of the doctor-patient relationship and communication: Karl Köhle was not a missionary who wanted to spread his worldview or his psychotherapeutic identity as a psychoanalyst to the world, but rather a benevolent mediator of a medicine oriented towards the person concerned, who paid very close attention to how he could reach learners with what was important to him. The same combination of creative and pragmatic elements characterised his involvement in the Carl Gustav Carus Foundation for Psychosomatic Medicine, of which he was President of the Foundation Board for many years. He and the Foundation particularly benefited from the fact that he was an excellent networker who could give you the impression on the phone that he had all the time in the world. Whenever a question arose in the Foundation Board, he knew someone who was interested in it at the moment and could help.

The word was always his companion and interaction and interpretation were important to him. So, Christoph Stosch remembers that one day he said to him, “No, you didn’t run me over, I said you stepped on my toes.” Now and then, he says, he still sits brooding over this semantic differential. In view of loud, undifferentiated debates of the present time, such a meticulous exegesis of what is said seems almost quirky, or more topical than ever. 

Karl Köhle was always interested in the persons sitting in front of him, be they patients, students or colleagues. He was restrained and yet clear and unambiguous in his observations and opinions. We will keep Karl Köhle in our hearts. Our heartfelt sympathy goes out to his family, and our honourable remembrance to him.

## Competing interests

The authors declare that they have no competing interests.

